# The Effectiveness of Herbal Versus Non-Herbal Mouthwash for Periodontal Health: A Literature Review

**DOI:** 10.7759/cureus.27956

**Published:** 2022-08-13

**Authors:** Samiksha Tidke, Gaurav Kumar Chhabra, Priyanka P Madhu, Amit Reche, Saee Wazurkar, Shriya R Singi

**Affiliations:** 1 Department of Public Health Dentistry, Datta Meghe Institute of Medical Sciences, Wardha, IND; 2 Epidemiology and Public Health, National Institute of Medical Sciences Dental College and Hospital, Jaipur, IND

**Keywords:** periodontitis, gingival inflammation, dental plaque, herbal mouthwash, non-herbal mouthwash

## Abstract

Dental plaque is a biofilm of microorganisms that present naturally on the exposed tooth surface; it is the main etiological factor for many periodontal conditions and other oral health issues and its regular removal from the oral cavity can prevent many periodontal problems. Despite several experiments using herbal oral care products to reduce dental plaque or gingivitis, the findings remain inconclusive. We performed a systematic literature search on PubMed and Cochrane Library for randomized controlled trials (RCTs) dating from 2001 up to and including the year 2021. The keywords and Medical Subject Headings (MeSH) terms comprised combinations of the following: herbal, clove oil, peppermint oil, ginger, basil, ajwain, betel leaf extract, neem, lavender, non-herbal, chlorhexidine, fluorides, hydrogen fluoride, hydrogen fluoride, stannous fluoride, and mouthwashes. Each of the titles that the search elicited was screened and duplicates were removed from the gathered results. The full-text versions of the remaining articles were downloaded and examined by title and abstract. Handsearching was not carried out. We initially identified 21 studies; 14 studies, which did not fulfill the selection criteria, were excluded. All the included studies reported a reduction in plaque index (PI) and gingival index (GI) scores in both herbal and non-herbal groups. Two studies reported the superiority of the non-herbal mouthwash over the herbal one while five of the studies showed no significant difference in PI and GI scores between herbal and non-herbal mouthwash, implying equal efficacy of both, i.e., Triphala, aloe vera, tea tree, and polyherbal groups like Zingiber officinale, Rosmarinus officinalis, and Calendula officinalis, and chlorhexidine. Current research suggests that herbal mouthwashes are as effective as non-herbal mouthwashes for reducing dental plaque in the short term; however, the evidence is based on low-quality trials.

## Introduction and background

Dental plaque is a biofilm of microorganisms that present naturally on the exposed tooth surface. It causes many periodontal conditions and other oral health issues and its regular removal from the oral cavity can prevent many periodontal problems. Plaque control normally involves preventive measures aimed at removing dental plaque and preventing it from recurring [1]. This can be accomplished either mechanically or chemically, and sometimes these two procedures are combined. With regular brushing, plaque can be removed from the tooth surface mechanically. Though toothpaste plays a very small role in its removal, the removal of bacterial plaque biofilm prevents gingivitis, periodontitis, and dental caries. To generate this inhibitory effect on plaque formation, a variety of compounds, primarily antimicrobial agents, have been added to dentifrices. Agents like chlorhexidine and triclosan have been shown to be effective [2-3]. Chlorhexidine is a broad-spectrum antimicrobial biguanide that has the strongest anti-plaque properties [4]. However, it is not recommended for long-term daily usage because it has been linked to a number of local side effects, which include brownish staining of the teeth, restorative materials, and the dorsum of the tongue [5]. In addition to interference with taste, periodontitis and dental caries prevention have traditionally relied on controlling biofilm formation on teeth [6]. Chemical plaque control using mouthwash is an adjuvant therapy that helps to remove plaque and prevent the build-up of microbiological plaque, potentially reducing the need for mechanical oral hygiene [7]. The use of "herbal" medicine has sparked interest and resulted in the emergence of complementary and alternative therapies in healthcare promotion in many regions of the world due to the increased awareness of indigenous medical traditions. Herbal compounds have been used in oral care products for some time, most commonly in South Asian nations, to help individuals with gingivitis improve their oral hygiene [2,8]. However, despite a vast number of experiments using various types of herbal mouthwash to reduce dental plaque or gingivitis, the findings remain inconclusive.

## Review

Materials and methods

Search Strategy 

A literature search was performed on PubMed and Cochrane Library for randomized controlled trials (RCTs) dating from 2001 up to and including the year 2021. The keywords and Medical Subject Headings (MeSH) terms comprised combinations of the following: herbal, clove oil, peppermint oil, ginger, basil, ajwain, betel leaf extract, neem, lavender, non-herbal, chlorhexidine, fluorides, hydrogen fluoride, hydrogen fluoride, stannous fluoride, and mouthwashes. Each of the titles that the search elicited was screened and duplicates were eliminated from the gathered results. The full-text versions of the remaining articles were downloaded and examined by title and abstract. Handsearching was not carried out.

The inclusion criteria were as follows: studies including patients without any systemic disease, both male and female patients, intervention: studies that included herbal mouthwashes, comparison: studies that included non-herbal mouthwashes, only RCTs, articles published from 2001 to 2021 (till July) in the English language, and articles from PubMed and Cochrane databases. The outcome measures were a reduction in the level of dental plaque and gingival inflammation. The outcomes were assessed on the following basis for both control and intervention arms: firstly, the mean reduction in plaque index (PI) based on Silness-Löe plaque index or modified Quigley-Hein or Turesky-Gilmore-Glickman modification of Quigley-Hein plaque index; secondly, the mean reduction of the gingival inflammation by Silness-Löe gingival index and, lastly, short term effects (studies up to one month).

The exclusion criteria were as follows: patients above the age of 50 and below the age of seven years, patients who underwent any oral prophylactic procedure before and within the study duration, articles published before 2001, articles in languages other than English, articles other than RCTs, patients on antimicrobial therapy during the procedure or 15 days prior to the treatment.

The aim of this literature review is to investigate and compare the effectiveness of herbal mouthwashes with non-herbal mouthwashes in controlling plaque and gingivitis.

Table 1 provides a summary of the included studies.

**Table 1:** Summary of included studies MQH: modified Quigley-Hein index; PI: plaque index; GI: gingival index; OHI: oral hygiene index; GBI: gingival bleeding index; MGI: modified gingival index; TRP: Triphala

Study	Country	Participant characteristics	Intervention group		Comparison group		Duration of outcome evaluation	Outcome
			Tools	Frequency	Tools	Frequency		
Bhat et al., 2014 [9]	India	72 individuals between 18-24 years of age including both male and female participants	MQH, GI	10 mL of mouthwash, twice a day for 1 minute after toothbrushing for a period of 4 weeks (1 month)	MQH GI	10 mL of mouthwash, twice a day for 1 minute after toothbrushing for a period of 4 weeks (1 month)	Baseline after 1 month	In comparing three Groups A, B, and C, which consisted of 0.15% Guava mouth-rinse, chlorhexidine mouth-rinse, and distilled water (placebo) respectively, all Groups showed a gradual reduction in PI and GI scores and a significant difference in all the test Groups from baseline to 3rd month. PI score was found to be in the high range at baseline and then showed a statistically significant reduction in all the Groups in the 1st month. GI score also showed significant change at the 1st and 3rd-month recall intervals. At the end of the third month, the GI score of Groups A and B was significantly higher than that of Group C
Pradeep et al., 2016 [10]	India	90 individuals around 25 years of age including both male and female participants	PI, GI, OHIS	15 mL of mouthwash, two times a day, 30 to 45 minutes after brushing. (additional instruction: after rinsing with mouthwash, do not rinse or eat for 30 minutes)	PI GI OHIS	15 mL of mouthwash, two times a day, 30 to 45 minutes after brushing. (additional instruction: after rinsing with mouthwash, do not rinse or eat for 30 minutes)	Baseline 7 days, 30 days, and 60 days	In comparing three Groups I, II, and III, all three Groups had a steady decline in PI and GI readings. At all-time intervals, there was a significant decrease in PI and GI scores in Groups II and III. In comparison to Group II (TRP Group) and Group III (CHX Group), there was a substantial difference in PI and GI reduction in Group I (placebo Group)
Kamath et al., 2019 [11]	India	152 individuals between 8-14 years of age including both males and females (Group 1: aloe vera mouthwash, Group 2: CHX, Group 3: tea tree, Group 4: placebo)	PI, GI	10 ml of mouthwash, twice daily for 30 seconds, once after lunch and once after dinner (additional instruction: refrain from eating, drinking, or rinsing the mouth for 30 minutes)	PI GI	10 ml of mouthwash, twice daily for 30 seconds, once after lunch and once after dinner (additional instruction: refrain from eating, drinking, or rinsing their mouth for 30 minutes)	Baseline 4 weeks, 2 weeks after stoppage of habit	In 3 Groups (aloe vera, CHX, and tea tree) mean plaque score showed a highly significant reduction as compared to the placebo Group (p<0.001), between baseline and 4 weeks of mouth-rinse. The mean gingival score showed a highly significant reduction in Groups 1, 2, and 3 as compared to Group 4 (p<0.001), between baseline and 4 weeks of mouth rinse
Nayak et al., 2019 [12]	India	60 patients aged between 18 and 40 years. Both male and female participants were included	MQH, GI	10 ml of mouth-rinse with an equal quantity of dilution for 1 minute was advised to be used two times daily 30 minutes after toothbrushing for a period of 30 days	MQH GI	10 ml of mouth-rinse with an equal quantity of dilution for 1 minute was advised to be used two times daily 30 minutes after toothbrushing for a period of 30 days	Baseline after 1 month, 3 months	Guava leaf extract mouth-rinses provided benefits until the end of the study, indicating that it could be useful as a supplement to professional oral prophylaxis. Despite being not as potent as the chemical constituent (0.2% chlorhexidine mouth-rinse), guava mouth-rinse outscored the placebo Group in terms of antimicrobial activity. Guava leaf extract mouth-rinse (Group 1) provided benefits until the end of the study period; it can now be used as a supplement to professional oral prophylaxis. Despite its lower potency, than the chemical constituent (0.2% chlorhexidine mouth rinse) Group 2, guava mouth-rinse outperformed placebo in terms of antimicrobial properties
Southern et al., 2015 [13]	India	152 individuals between 20 and 50 years of age; both male and female participants were included	PI, GI	15 ml of mouth-rinse for 30 seconds twice a day	PI, GI	15 ml of mouth-rinse for 30 seconds twice a day	Baseline after 3 weeks	When gingival index scores and plaque index scores were compared for baseline parameters, Group I (herbal) did not show statistically significant differences from Group II (peridex)
Mahyari et al., 2015 [14]	Iran	60 patients participated and were divided into three groups (Group 1: polyherbal mouthwash, Group 2: chlorhexidine mouthwash, and Group 3: placebo mouthwash)	MGI, GBI, MQH	Twice a day for 30 seconds (after breakfast and dinner) for 14 days	MGI, GBI, MQH	Twice a day for 30 seconds (after breakfast and dinner) for 14 days	Baseline 7 days, 14 days	There were statistically significant improvements in efficacy measures i.e. MGI, GBI, and MQH scores from baseline to 14 days in polyherbal as well as chlorhexidine mouthwash Groups; however, the scores remained statistically unchanged in the placebo group
Jalaluddin et al., 2017 [15]	India	40 individuals between 18-35 years of age	PI, GI	Group I received 10 mL of chlorhexidine gluconate mouthwash and was directed to rinse for 1 minute, while Group II received 10 mL of neem mouthwash and was instructed to rinse for 15 days	PI, GI	Group I received 10 mL of chlorhexidine gluconate mouthwash and was directed to rinse for 1 minute, while Group II received 10 mL of neem mouthwash and was instructed to rinse for 15 days	Baseline 15 days	There was a statistically signiﬁcant difference in both Groups at baseline and after the intervention. There was a slight reduction of plaque level in the neem Group compared with the chlorhexidine mouthwash group. Both Groups' GI recordings were reduced, whereas only the baseline scores showed a statistically significant difference

Methodology

**Figure 1:**
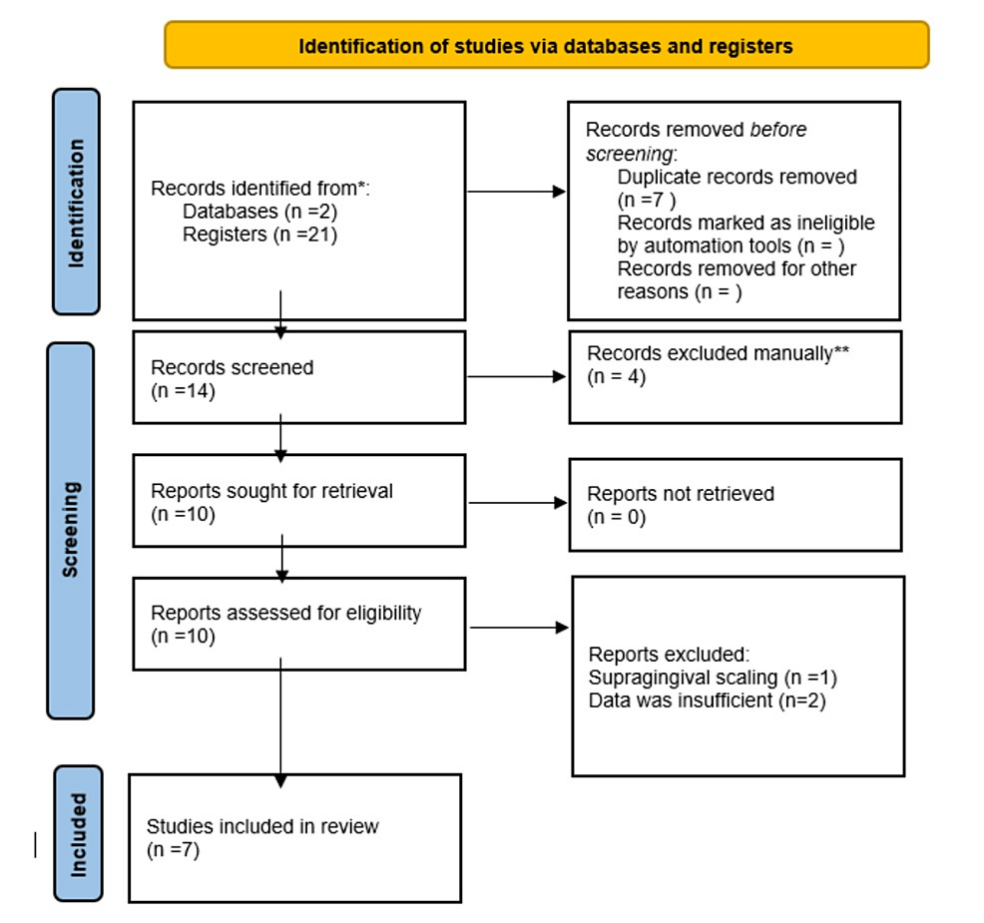
Methodology* *[16]

The study methodology is illustrated in Figure 1.

Results

Plaque is the main etiological factor leading to gingivitis and periodontitis. Daily plaque removal is important for gingival health. With regular brushing, plaque can be removed from the tooth surface mechanically. However, toothpaste plays a very small role in its removal. Removal of microbial dental plaque biofilm is a must as it prevents gingivitis, periodontitis, and dental caries, and for this purpose, a variety of compounds, primarily antimicrobial agents, have been added to dentifrices and mouthwashes. Our search strategy initially yielded 21 articles; the scrutiny of titles and abstracts reduced the number of articles to 14. Studies that did not meet the selection criteria were excluded. Seven RCTs were included in the review, which comprised 626 patients, based on the inclusion criteria. A summary of included studies is shown in Table 1.

An intention-to-treat analysis was performed. When published data was missing, incomplete, or inconsistent with RCT protocols, we contacted the authors/manufacturers for more information. If studies did not have outcome measures of interest, did not specify randomization or intention-to-treat analysis, or had missing data, we contacted authors through email. Clinical heterogeneity was seen in the herbal ingredients present in mouthwash in RCTs. All the studies showed a significant reduction in both herbal and non-herbal groups, but the difference in PI and GI scores between the herbal and non-herbal groups was insignificant. Two of the studies (those by Southern et al. and Nayak et al.) showed that the herbal group did not yield a statically significant decrease in PI and GI scores compared to the non-herbal group, indicating the superiority of the non-herbal group. Herbal ingredients like Triphala, aloe vera, tea tree, and polyherbal groups like Zingiber officinale, Rosmarinus officinalis, and Calendula officinalis were found to be equally effective than non-herbal counterparts. Data were insufficient to ascertain as to which mouthwash is more potent in reducing plaque and gingivitis although both groups were found to be equally effective and the difference in the values was insignificant.

Discussion

It was observed that despite brushing twice daily, which removes the main etiological factor leading to gingivitis and periodontitis, these diseases are common in a majority of people [17]. Maintaining an acceptable degree of plaque control using traditional mechanical methods and dentifrices is certainly difficult, but it is now the only feasible means of improving periodontal health on a mass level from a therapeutic standpoint [1]. The majority of the plaque is mechanically removed; however, there is still thin dental plaque that can be easily removed chemically. Hence, the current study recommends a combination of chemical and mechanical oral hygiene treatments for the most effective plaque removal [17]. When compared to other potential antiplaque agents, chlorhexidine has been shown to have the highest success rate and is thus recognized as a gold standard for plaque removal. But because of its local side effects, such as extrinsic stains and taste irregularities, chlorhexidine's long-term use has been limited. The use of CPC-containing mouth-rinse as an adjuvant to toothbrushing has been demonstrated to be successful in reducing dental plaque and gingival irritation over the long and short term. Because Ayurvedic medicinal plants have no or minimum negative effects, they are employed in a variety of treatments.

As part of the search for a suitable adjuvant to mechanical therapy for long-term usage, some herbal mouthwash and herbal extracts have been evaluated both in vitro and in vivo [18]. It has been seen that aloe vera is a potent antimicrobial agent and can be used for plaque removal. It can act as a good herbal substitute as it overcomes side effects such as immediate hypersensitivity, toxicity, and tooth staining. In a study by Kamath et al. [11], aloe vera mouthwash was compared with chlorhexidine mouthwash, and it was found to be equally effective as chlorhexidine. The study conducted by Bhat et al. demonstrated the plaque-preventing potential of herbal mouthwashes, which include ingredients like S. persica, P. betle, T. Billerica, and E. cardamomum. S. persica, a toothbrush tree locally called “Miswak”, has been proven to be an efficient antiplaque agent in numerous studies [9]. When compared to chlorhexidine mouthwash, herbal mouthwash was found to be just as effective at reducing plaque and gingivitis [9]. In a study conducted by Pradeep et al., TRP mouthwash was found to reduce inflammatory markers, resulting in gingivitis improvement [10]. The findings were comparable to those related to CHX mouthwash, which has long been considered the "gold standard" in the treatment of gingivitis and periodontitis. Hence, TRP mouthwash may be regarded as a possible therapeutic agent in the treatment of gingivitis. Triphala mouthwash appears to have the same clinical efficacy as CHX-MW in improving plaque-induced gingivitis. TRP is a cost-effective option that is readily available and has minimal side effects on periodontal tissues [19]. Mahyari et al. found satisfactory changes in gingival and plaque scores from baseline to day 14; however, the scores remained statistically unchanged in the placebo group [14]. Polyherbal mouthwash was found to be safe and effective in reducing plaque and gingivitis. In the study performed by Southern et.al., it was found that chlorhexidine was the only rinse to demonstrate a statistically significant effect in the reduction of mean GI and PI scores. Chlorhexidine was more effective in reducing plaque and gingival scores when compared to herbal and placebo [13]. A study performed by Jalaluddin et al. stated that in both herbal and chlorhexidine groups, there was a reduction in PI and GI scores, but a significant difference was only seen in baseline scores [15].

## Conclusions

Extensive research has been conducted to determine the effects of chlorhexidine and herbal mouthwashes individually, but there is very limited data comparing their efficacy both clinically and experimentally. Herbal toothpaste seems to be as effective as non-herbal toothpaste, but it does not surpass the effectiveness of fluoride toothpaste. Some of these substances can lead to undesirable side effects such as tooth staining and altered taste. As a result, natural components in herbal dentifrices have received more attention. Ayurvedic medicine takes a holistic approach to treating humankind. It can maintain a balance between general and oral health, as well as the environment, which is critical for human well-being in today's world. Consequently, plaque control should include both chemical and mechanical methods.
